# Abortion, from the Standpoint of Personal Experience

**Published:** 1886-12

**Authors:** S. H. Stout

**Affiliations:** Cisco, Texas


DANIEL'S

### Texas Medical Journal.

PUBLISHED MONTHLY.—SUBSCRIPTION $2.00 A YEAR.

Vol. 2. AUSTIN, DECEMBER, 1886. No. 6.

### ORIGINAL ARTICLES.

CONTRIBUTED EXCLUSIVELY TO THIS JOURNAL.

*The Articles in this Department are accepted and published with the understanding that we are not responsible for, nor do we indorse the views and opinions of the writers by so doing.*

### ABORTION.

FROM THE STAND-POINT OF PERSONAL EXPERIENCE.

*By S. H. Stout, A. M., M. D., L. L. D., Cisco, Texas.*

For Daniel's Texas Medical Journal.

UPON the intelligent and conscientious obstetrician, threatened abortion and the management of inevitable miscarriages, often inflict the most worrying anxiety. Abortion is an accident of the gravest import to the child-bearing female, for it often seriously compromises her bodily health and mars her happiness.

In attempting here to record an outline of individual experience and the conclusions founded thereon, it is not proposed to enter into a discussion of the physiology of gestation and parturition, nor largely to descant upon the pathology of miscarriages.

In my experience, unwonted muscular effort has been the most frequent exciting cause of abortion. Stooping and reaching up while using muscular efforts for a considerable length of time are attitudes most frequently provocative of this disaster; in the former, fatiguing muscular effort either in lifting or pushing; in the latter, long and tiresome work in tacking or tying curtains overhead. Great bodily fatigue and mental anxiety in nursing the sick, in my experience, has also been a frequent exciting cause of abortion.

I have also known females, descended from a common stock, much given to aborting. In one family of tour sisters every one aborted from one to three times as often as they carried a fœtus to full term. In one family of the highest respectability every married female for two generations, had miscarried one or more times. The flooding in their cases was fearful in quantity. In every case the placenta was diseased. Having found a son of one of these ladies covered from birth with copper colored splotches, through his father, who was a very sensible and discreet man, I set on foot an enquiry as to the family history. In the fourth generation antecedent to that of his son, he found the maternal ancestor was not respectable as to virtue. During three generations descended from her, there was not a stain upon the character of any female of her stock. Indeed they were all respectable and occupied social positions of influence. Anti-syphilitic remedies cured the boy, and thus I had proof strongly corroborating the suspicion previously entertained, that the family for several generations had suffered from hereditary syphilis.

Falling, being thrown from a height, fright, jumping, running; attacks of fever, diarrhœa, gastritis, pneumonia and other phlegmasia, I have known to produce abortion.

During one epidemic of measles I attended upon three females pregnant for four, five, and seven months respectively, neither of whom aborted.

I have never happened to treat any female pregnant, laboring under any one of the other exanthemata.

In only one case have I ever had to produce artificial abortion to save the mother's life, threatened by persistent and uncontrollable nausea and vomiting of pregnancy. I remember no case of abortion produced by the nausea and vomiting of pregnancy. In two instances abortion supervened upon attacks of acute gastritis. When the nausea of pregnancy, however worrying it may be to the patient, does not seriously threaten death from inanition, I am inclined to the opinion that it is protective, rather than a serious injury to the mother and her fœtus, whenever the manufacture of plastic blood is in excess of their needs or power of appropriation. I have met with numerous mothers, who suffered from this sickness during the first five or six months of pregnancy, and vomited daily half, or more than half, of the food they ingested, that went on fattening until parturition at full term, giving birth to fat babies. Robust, hearty, laboring women, puking every day more or less often, have been known to attend to their daily duties, maintaining otherwise good bodily health, and jolly spirits, to the end of the term of gestation.

The nausea of pregnancy has oftenest given me trouble among society women, with whom any, the least ailment or circumstance, that interrupts their social enjoyments or engagements or ambition, is a source of continual worry, and consequent persistent appeals for relief.

In all cases of threatened abortion, a careful investigation should be at once made, to ascertain whether the case is one of inevitable or preventable miscarriage. In either case the patient should be at once required to assume the recumbent posture and to persistently maintain it until in the latter instance the dangerous symptoms have subsided, and in the former, until the flow of blood has ceased and the uterine involution is so far complete as to forbid the provocation of metrorrhagia, a very common sequent of abortion.

In cases of preventable abortion, I have never found any article of the materia medica so efficient as opium, which I have generally administered in the form of a dry pill repeated p. r. n. When the stomach did not tolerate the medicine, an injection or suppository per anum has often proven efficient.

A mustard plaster three inches wide and six or seven inches long, placed over the lumbar region of the spine, I never fail to prescribe, for reasons obvious to any intelligent student of the pathology of abortion. I do not remember a case in which I have failed to arrest a preventable threatened abortion by the practice above outlined.

I do not regard an abortion to be preventable when the bag of waters has been ruptured or it is protruding unbroken through the external os uteri. The frequently accompanying hemorrhage becomes then the greater source of anxiety. The sooner the delivery of all the parts of the ovum is completed, the better in such cases for the patient, and for the practitioner. If the fœtus is in reach of the fingers it is my practice to aid its delivery by gentle traction, for it is desirable to get it out of the way as soon as the os is dilated enough to let it pass, in order that the delivery of the placenta may be accomplished, if possible, at once.

It is my experience that a retained placenta in cases of miscarriage can be more easily delivered immediately after the delivery of the fœtus than several hours afterwards. At that time the circular fibres of the neck of the womb are more flaccid and yielding to the fingers or to instruments, (if their introduction is necessary,) than several hours afterwards.

(Right here it is proper to remark that in all my obstetric practice I have never met with a case of hour-glass contraction of the uterus in births at full term).

If, after the birth of the fœtus, the practitioner does not take advantage of the relaxed condition of the circular fibres of the neck of the uterus, or rather of those surrounding the internal os, he will often have to resort to tents or other means of overcoming their contraction, using either his fingers or some one of the instruments used for stretching the os; and even then, he is liable to find he has produced irritation of the organ, as manifested by its increased heat and tenderness, and the unyielding character of its circular fibres.

For more than thirty years it has been my practice to use the wire loop, and no other instrument whatever, when the fingers cannot effect the delivery of the placenta in early abortions. I am not aware that any one ever before myself thus used the wire loop. Certain it is, its use was not suggested to me by any one. I first resorted to it because it is as reasonably an appropriate instrument for drawing out a placenta from the cavity of the womb, as for drawing a cork from the cavity of a bottle, due allowance being made for the different nature of a hollow living organism, and an inorganic vessel.

In delivering a retained placenta in abortions prior to the sixth month of gestation, I always use a wire so flexible that I can shape it without the aid of the vise or forceps. Only in cases further advanced would I use an instrument not easily flexible with the hands of the operator. In a majority of cases, in which I have resorted to the wire loop, I have found the wire on the premises of the patient, and there improvised the instrument. In very early abortions, the wire with which the straw and handle of the common house broom are bound together, admirably answers the purpose. If it proves too flexible it can be doubled and twisted together before forming the loop. I make the loop as follows: A piece of wire from sixteen to twenty inches in length is doubled upon itself at or near the middle, where the loop is formed. From two to three inches from the middle the two extremities of the wire are twisted upon each other, and at their ends turned up so as to form a guide to the direction of the pelvic curve of the instrument. The two extremities of the wire thus twisted upon themselves, form a handle for the instrument, which, exclusive of the loop, ought to be from eight to sixteen inches long. All of the handle behind the loop I cover with cloth or adhesive plaster to prevent the catching of any of the soft parts of the mother in the interstices of the twisted wire. This loop can, with caution, be safely introduced into the cavity of the womb. If while there, it is cautiously rotated, more or less of the placenta becomes engaged in the loop, when, by gentle traction on the instrument its delivery is wholly or in part effected. Should the whole placenta fail of delivery at the first introduction and withdrawal of the instrument, it should be again and again (if need be) introduced until there is reasonable ground to believe that all of the mass has been delivered. The wire loop, before introduction into the womb, should be disinfected and annointed.

Too much caution as to remaining in the recumbent posture until involution of the uterus has been completed, cannot be given a female who has aborted. Generally a patient who has aborted should remain in bed as long as should a mother giving birth to a child at full term.

In every case of abortion I have ever attended I have been able without serious difficulty to deliver the placenta, save in a single case which I met with prior to my use of the wire loop.

I was once called to see a woman suffering from septicæmia consequent upon retained placenta after an abortion, about ten days prior to my visit. She died. I have had so little difficulty in delivering the placenta after abortion that I take no interest in the discussion as to whether it is safer to leave its delivery to nature or to aid it by art..

The case of septicæmia above mentioned was met with early in my professional career, and has ever stimulated me to deliver the placenta as soon as possible.

The woman who has once aborted is very liable to a repetition of the accident at about the same period of gestation at which the first abortion occurred. A young married woman aborted in four pregnancies between the fifth and sixth month, before applying to me for advice. In addition to the usual precautions, I directed her to apply a blister three by six inches over the spine, in the lumbar region, immediately upon the occurrence of the least pain in that part, and to go to bed and remain there, if need be, for at least a month. Following this advice during the next pregnancy she carried the fœtus to the full term. The habit of aborting was broken up. She was, when I last saw her, the happy mother of five healthy children, having never again aborted, or as she asserted, been threatened with abortion, during any subsequent pregnancy.

